# Two New Genera and Species of the Parasitic Copepod Family Chondracanthidae Milne Edwards, 1840 (Copepoda: Cyclopoida) from Deep-Sea Fishes Off Suruga Bay, Japan

**DOI:** 10.1007/s11686-024-00820-3

**Published:** 2024-03-11

**Authors:** Panakkool Thamban Aneesh, Susumu Ohtsuka, Yusuke Kondo, Ameri Kottarathil Helna

**Affiliations:** 1 Blue Innovation Division, Seto Inland Sea Carbon Neutral Research Center, 5-8-1 Minato-Machi, Takehara, Hiroshima 725-0024 Japan; 2 Travancore Nature History Society (TNHS), MBRRA, Mathrubhumi Road, Vanchiyoor, Trivandrum, Kerala 695035 India; 3 Universiti Sultan Zainal Abidin, Gong Badak Campus, 21300 Kuala Terengganu, Terengganu Malaysia; 4 Regional Forensic Science Laboratory, Kannur, Kerala 670002 India

**Keywords:** *Avatar nishidai***gen.** et **sp. nov.**, Chondracanthids, Copepoda, Fish parasite, *Kokeshioides surugaensis***gen.** et **sp. nov**

## Abstract

**Purpose:**

The present paper describes two new genera and species of the parasitic copepod family Chondracanthidae Milne Edwards, 1840 based on specimens collected from two species of deep-sea fishes at a depth of 212 m off Suruga Bay, Japan. *Avatar nishidai* **gen.** et **sp. nov.** is described from the host fish *Chaunax abei* Le Danois, 1978 (Chaunacidae). *Kokeshioides surugaensis* **gen.** et **sp. nov.** is described from the host fish *Setarches longimanus* (Alcock, 1894) (Setarchidae).

**Methods:**

Fresh specimens of chondracanthids were collected from the buccal cavity of two species of deep-sea fishes (fish hosts were frozen), *Chaunax abei* Le Danois, 1978 (Lophiiformes: Chaunacidae) and *Setarches longimanus* (Alcock, 1894) (Perciformes: Setarchidae), caught at a depth of 212 m in Suruga Bay, Japan (34° 37′48.87″ N, 138° 43′2.958″ E). Both the species are described and illustrated based on ovigerous females.

**Results:**

The genus *Avatar* **gen. nov.** can readily be distinguished from all other chondracanthid genera by the following combination of features: cephalothorax slightly wider than long with anterior pair of large and posterior pair of small lateral lobes, and two pairs of ventro-lateral processes; the very posteriormost part of the first pedigerous somite contributes to the neck; cylindrical trunk with two pairs of blunt proximal fusiform processes; antennule with small knob terminally; antenna bearing distal endopodal segment; labrum protruding ventrally; two pairs of biramous legs each with 2-segmented rami. *Kokeshioides* **gen. nov.** has the following combinations of features that distinguish it from other chondracanthid genera: body flattened, without lateral processes; cephalothorax much wider than long, with paired anterolateral and posterolateral lobes, folded ventrally; the very posteriormost part of the first pedigerous somite contributes to the neck; mandible elongate; legs unique, heavily sclerotized, represented by two pairs of acutely pointed processes.

**Conclusion:**

With the addition of two new genera presently reported, the family Chondracanthidae currently includes 52 valid genera. Among the described genera *Avatar* **gen. nov.** seems to be very primitive, while *Kokeshioides* **gen. nov.** is highly advanced. The deduced evolutionary history of chondracanthid genera is also discussed.

## Introduction

Studies on parasitic copepods infecting deep-sea fishes are still rare in comparison with those on shallow-water taxa. Deep-sea parasitic copepods are highly restricted to some genera belonging to the following families: Pennellidae Burmeister, 1835, Chondracanthidae Milne Edwards, 1840, Sphyriidae Wilson, 1919, Hyponeoidae Heegaard, 1962, and Hatschekiidae Kabata, 1979 (see Table 1 in Boxshall) [1].

**Table 1:** Character differences between the closely related chondracanthid genera, ***Avatar* gen. nov., *Kokeshioides* gen. nov.**, *Blias* Krøyer, 1863, *Diocus* Krøyer, 1863, *Humphreysia* Leigh-Sharpe, 1934, *Immanthe* Leigh-Sharpe, 1934, and *Juanettia* Wilson, 1921 collated from original descriptions and, where applicable, redescriptions (see Wilson 1921; Ho 1969, 1994)

Characters	*Avatar* **gen. nov**	*Kokeshioides* **gen. nov**	*Blias*	*Diocus*	*Humphreysia*	*Immanthe*	*Juanettia*
Antenna	With vestigial second endopodal segment	vestigial second endopodal segment absent	Terminal segment trifurcate	Terminal segment ‘T’ shaped	With vestigial second endopodal segment	Unknown	With vestigial second endopodal segment
Cephalic process	Present	Absent	Absent	Present	Present	Present	Present
Trunk processes	Present	Absent	Absent	Present	Absent	Absent	Present
Legs	Two pairs of biramous legs present, rami two-segmented	Two pairs of spiniform legs	Two pairs of modified, biramous, unsegmented legs. Lobate protopod and lobe-like rami	Two pairs of biramous rudimentary legs	One pair of unmodified legs	Two pairs of biramous rudimentary legs; endopod smaller than exopod	One pair of biramous leg, rami two-segmented

The Chondracanthidae is one of the most speciose copepod families, that utilize fishes as hosts accommodating nearly 192 species in 50 valid genera [2]. Among them, 27 genera are monotypic and only two genera have more than ten species, *Acanthochondria* Oakley, 1930 with 54 valid species, and *Chondracanthus* Delaroche, 1811 with 41 valid species [2–6]. Molecular analyses are absolutely needed to confirm the validity of monotypic genera.

The documentation of the parasitic copepod fauna in Japanese waters began with the significant contributions by Yamaguti [7], Yamaguti and Yamasu [8], Wilson [9, 10], Shiino [11, 12] and Izawa [13], followed by Ohtsuka *et al.* [14, 15], Uyeno and Nagasawa [16] and Nagasawa *et al.* [17]. The family Chondracanthidae is comparatively well documented in Japanese waters, with 48 valid species in 20 genera (see Nagasawa *et al.* [17]).

In the presently reported study, we describe two new genera and species collected from two species of deep-sea fishes in Suruga Bay, Japan.

## Materials and Methods

Specimens of chondracanthids were collected from the buccal cavity of two species of deep-sea fishes (fish hosts were frozen), *Chaunax abei* Le Danois, 1978 (Lophiiformes: Chaunacidae) and *Setarches longimanus* (Alcock, 1894) (Perciformes: Setarchidae), caught at a depth of 212 m in Suruga Bay, Japan (34°37′48.87″ N, 138°43′2.958″ E). Methods for preservation, dissection, mounting, and drawing of appendages were according to the techniques described in Aneesh *et al.* [18–21]. The specimens were microphotographed using Olympus microscopes (Olympus SZX7 and Olympus Bx50, Olympus Co., Ltd.) and image-capturing software (DP2-SAL, Olympus Co., Ltd). Total body length was measured (without egg sacs) from the anterior margin of the cephalothorax to the distal end of the caudal rami. Drawings were digital-inked using Adobe Illustrator and a WACOM CTL-472/K0-c drawing pad. Morphological terminology follows Huys and Boxshall [22]. The taxonomy and nomenclature of host fishes were adopted from Catalogue of Fishes [23] and FishBase [24]. The type material is deposited in the National Museum of Nature and Science, Tsukuba, Japan.

### Taxonomy

- **Order Cyclopoida Burmeister, 1834**
- **Family Chondracanthidae Milne Edwards, 1840**

### Genus ***Avatar*gen. nov.**

*Type species*: *Avatar nishidai* **gen.** et **sp. nov.** by original designation.

***Diagnosis based on the adult female*** (**bold = key features**). Body small, flattened. Cephalothorax and first pedigerous somite fused forming cephalothorax. Cephalothorax, slightly wider than long with anterior pair of large and posterior pair of small lobes, and two pairs of ventro-lateral processes; latter processes located at level of mouthparts. **The very posterior most part of the first pedigerous somite contributes to the neck**. Third and fourth pedigerous somites fused into cylindrical trunk, bearing two pairs of blunt proximal fusiform processes. Antennule with large, fleshy, basal and terminal portion with few small elements. Antenna uncinate, distal endopodal segment represented by atrophied tip of antenna, a slender segment with five elements. **Labrum protruding ventrally**. Mandible falcate and bilaterally denticulated. Maxillule armed with two elements. Maxilla 2-segmented, terminal segment curved, slender, and with attenuated process bearing row of teeth. Maxilliped 3-segmented, armature of the typical chondracanthid type. **Two pairs of biramous legs, each with 2-segmented rami**. **Other legs absent**. Genitoabdomen with paired genital apertures on ventral surface. Caudal rami small, bearing one long and three short elements. Egg sac multiseriate.

***Etymology***: The generic name is derived from a world-famous epic science fiction film, James Cameron’s “Avatar”, in which the dragon-like aerial predator “Mountain Banshee” with two pairs of wings reminds us of the present new taxon with two pairs of lateral processes on the trunk. Gender feminine.

***Remarks***: *Avatar* can readily be separated from all other known chondracanthid genera by the following combination of features: (1) cephalothorax, slightly wider than long with anterior pair of large and posterior pair of small lobes, and two pairs of lateral processes; (2) posteriormost part of the first pedigerous somite contributes to the neck; (3) cylindrical trunk with two pairs of blunt, proximally fusiform processes; (4) antennule with small terminal knob; (5) antenna distal endopodal segment represented by the atrophied tip, slender segment with three apical elements; (6) labrum protruding ventrally; (7) two pairs of biramous legs, each with 2-segmented rami.

The presence of biramous legs 1 and 2, each with 2-segmented rami and an accessory lobe on the antenna indicates that *Avatar* **gen. nov.** resembles other chondracanthid genera such as *Blias* Krøyer, 1863, *Diocus* Krøyer, 1863, *Humphreysia* Leigh-Sharpe, 1934, *Immanthe* Leigh-Sharpe, 1934, and *Juanettia* Wilson, 1921 (see Table 1). The genus *Blias* differs from *Avatar* **gen. nov.** by (1) terminal antennary segment trifurcate (vs. uncinate antenna with accessory process in *Avatar* **gen. nov.**), (2) body processes absent (vs. cephalic and trunk processes present), and (3) legs biramous, unsegmented, each with a lobate protopod and rami (vs. biramous legs present with distinctly 2-segmented rami). *Diocus* can be distinguished from *Avatar* **gen. nov.** by (1) antennary segment T-shaped (vs. uncinate antenna with accessory processes), and (2) biramous legs without clear segmentation (vs. rami distinctly 2-segmented). The monotypic genus *Humphreysia* differs from *Avatar* **gen. nov.** by (1) trunk processes absent (vs. present), and (2) only one pair of biramous, unsegmented legs (vs. two pairs). Another monotypic genus *Immanthe* differs from *Avatar* **gen. nov.** by (1) trunk processes absent (vs. present), and (2) rami of both legs with endopod smaller than exopod (vs. both rami equal in size). The new genus *Avatar* is most closely related to the relatively primitive chondracanthid genus *Juanettia*, but both can be separated from each other by the number of biramous legs and their segmentation: only one pair of biramous, 1-segmented legs in *Juanettia* (vs. two pairs with 2-segmented rami).

### ***Avatar nishidai*gen. et sp. nov.** (Figs. 1, 2, 3, 4, 5)

**Fig. 1:**
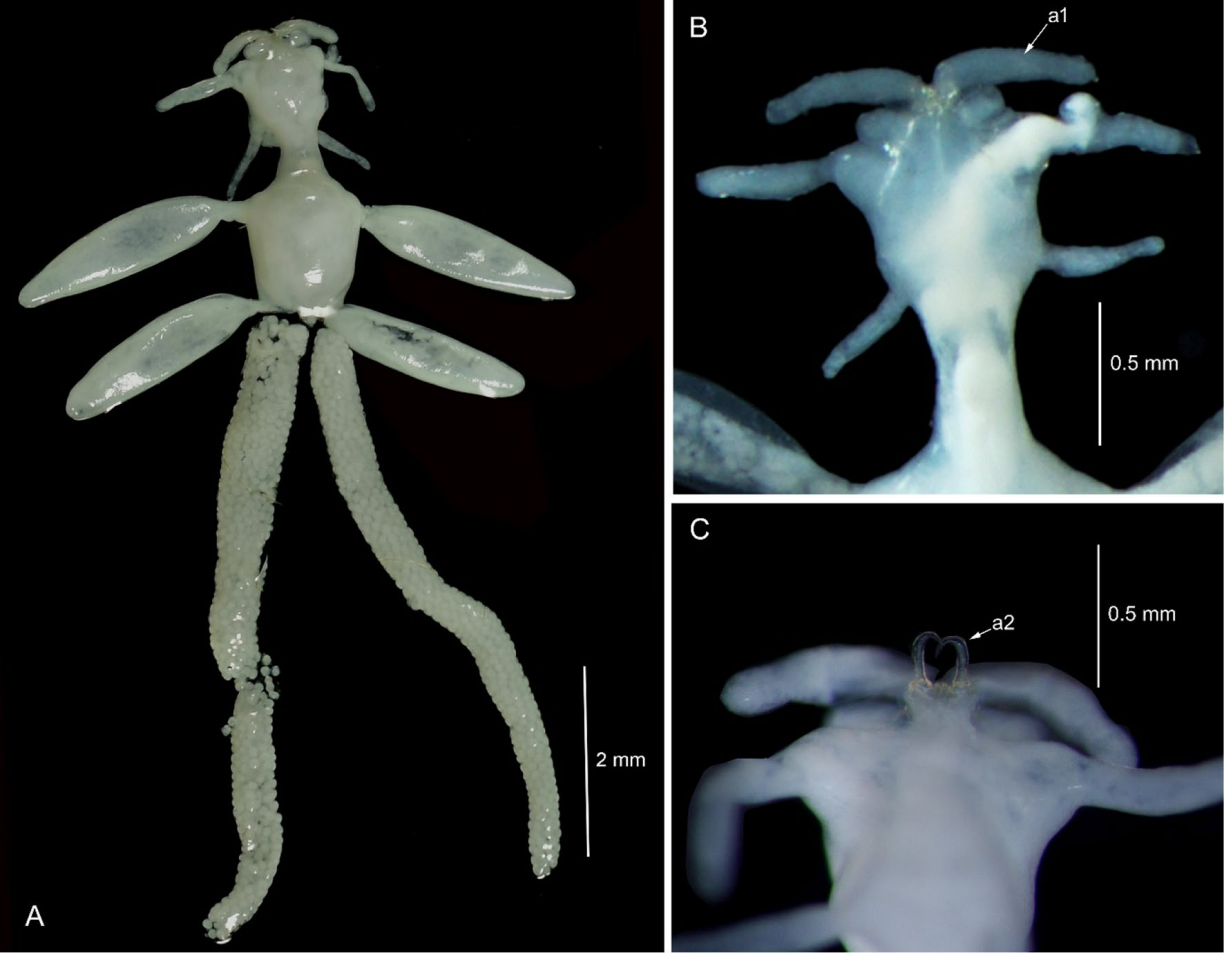
*Avatar nishidai* gen. et sp. nov., holotype female. A Dorsal view. B Cephalothorax dorsal view. C Cephalothorax ventral view. a1: antennule, a2: antenna

**Fig. 2:**
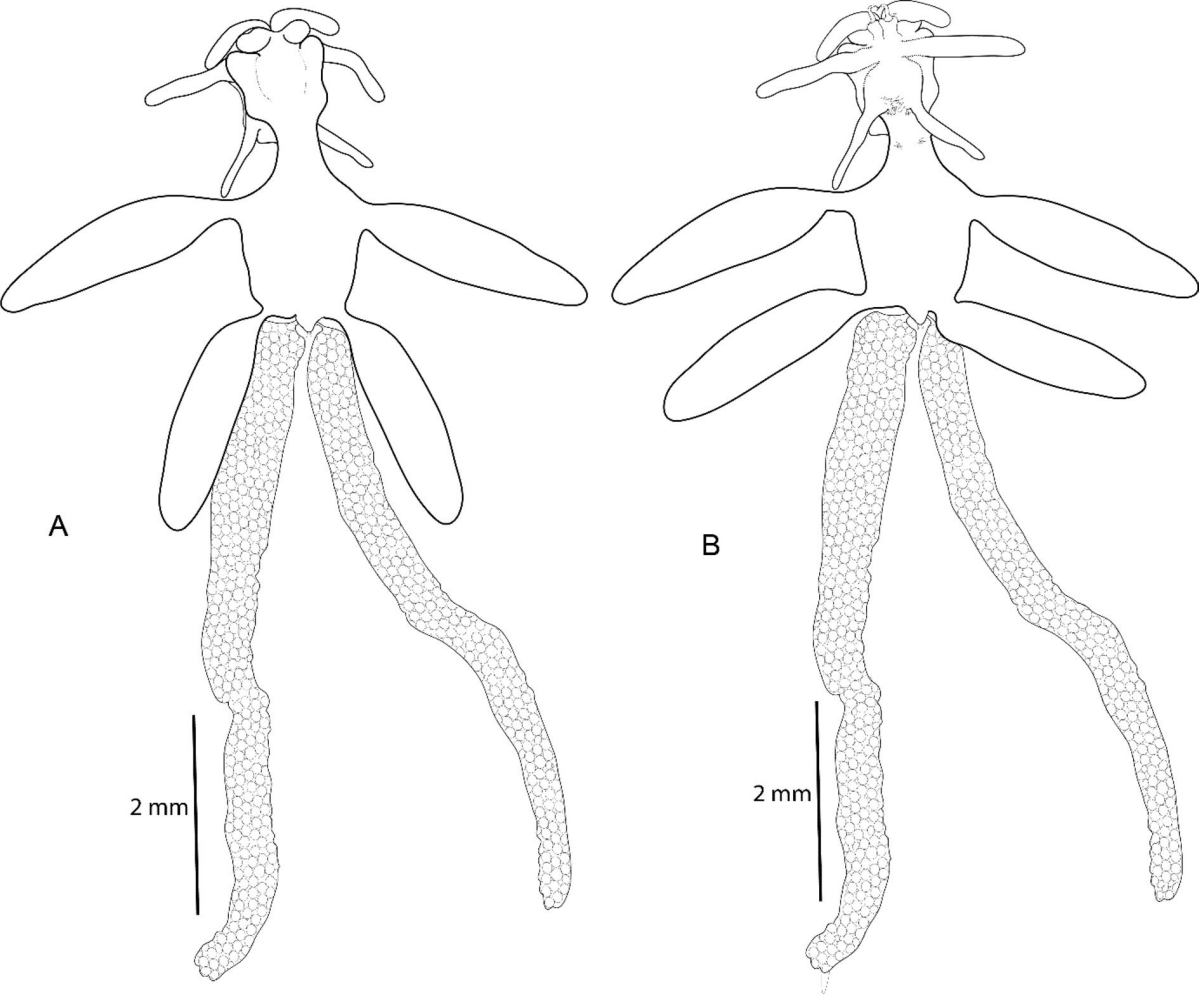
*Avatar nishidai* gen. et sp. nov., holotype female. A Dorsal view. B Ventral view

**Fig. 3:**
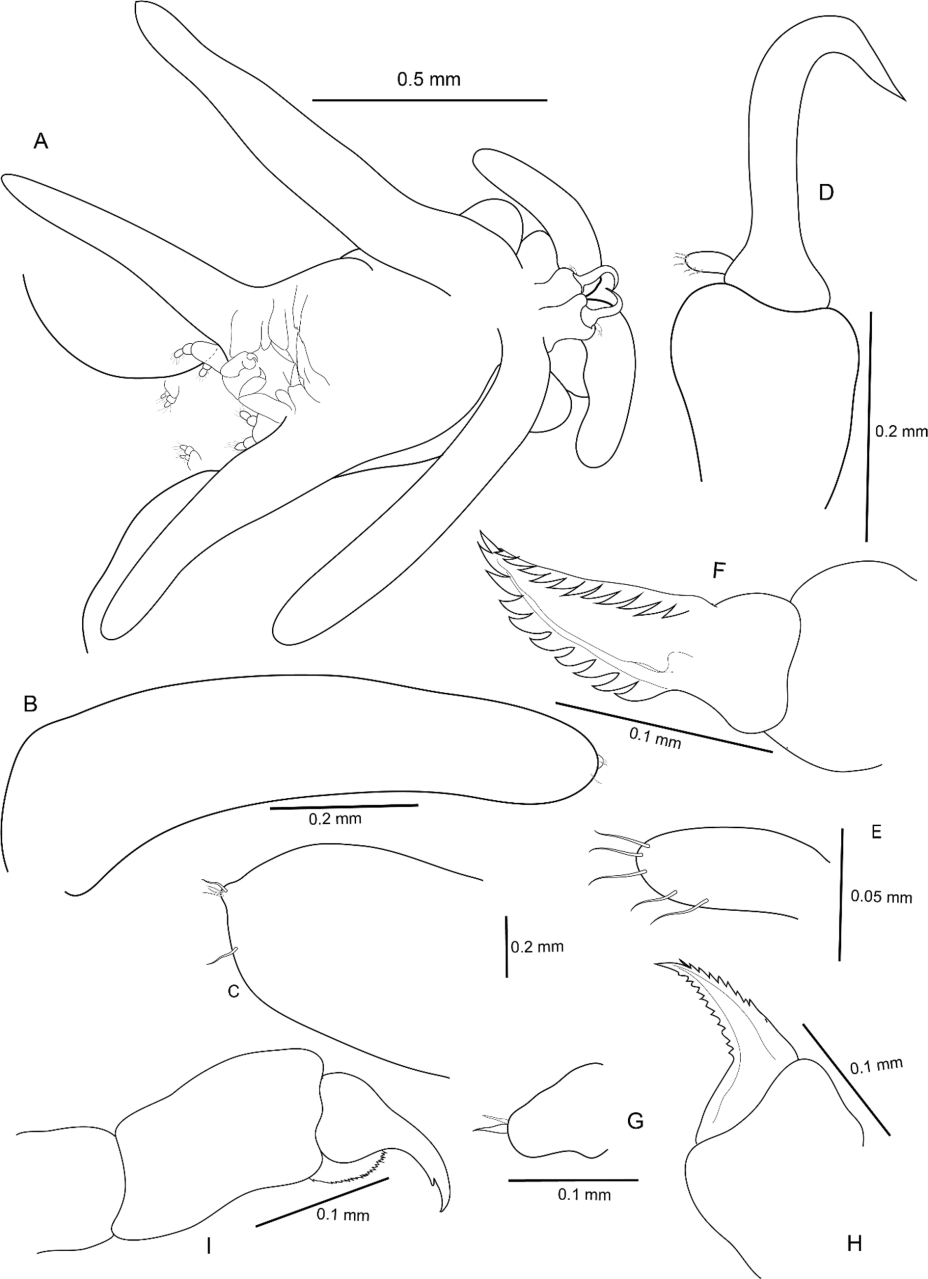
*Avatar nishidai* gen. et sp. nov., holotype female: A Cephalothorax ventral view. B Antennule. C Antennule apex enlarged. D Antenna. E Antenna distal endopodal segment, F mandible. G Maxillule. H Maxilla. I Maxilliped

**Fig. 4:**
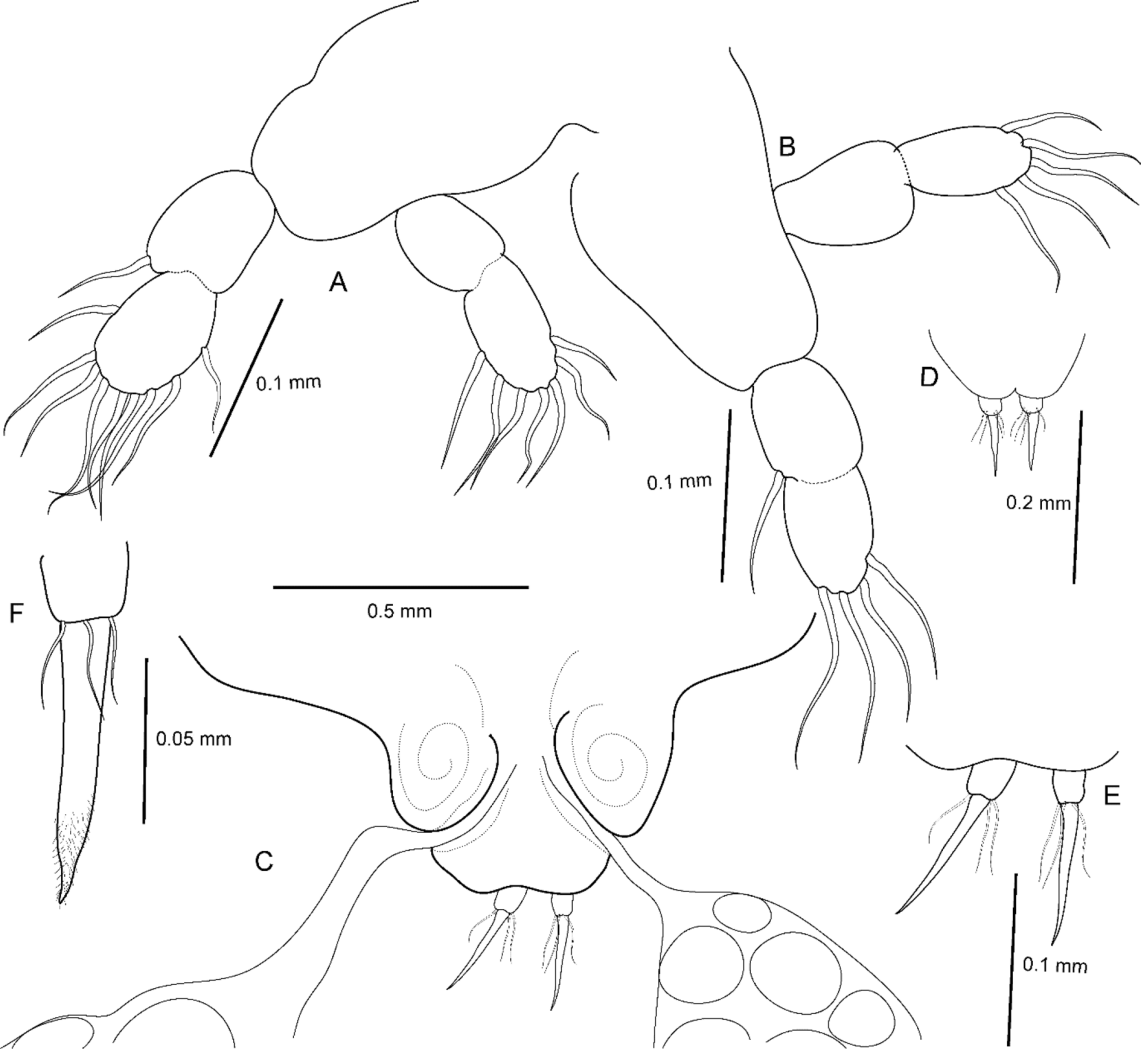
*Avatar nishidai* gen. et sp. nov., holotype female. A Leg 1. B Leg 2. C Genitoabdomen. D,E Caudal rami. F Tip of caudal ramus showing caudal setae

**Fig. 5:**
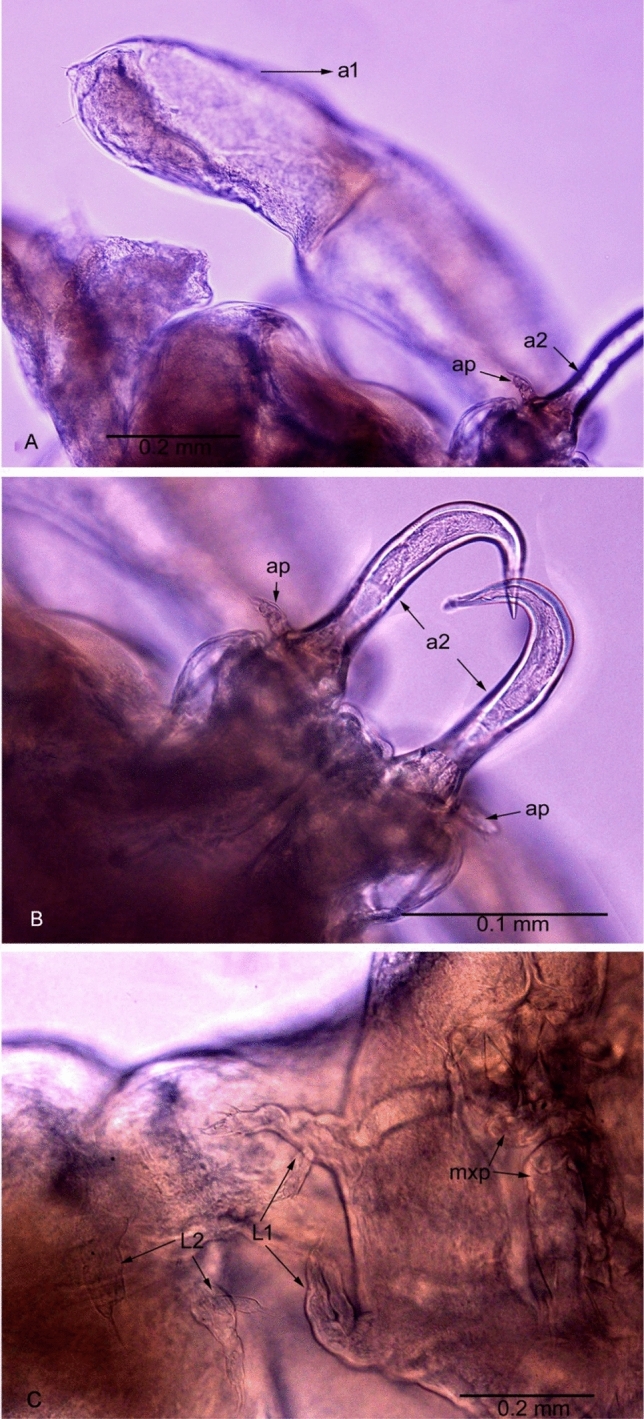
*Avatar nishidai* gen. et sp. nov. A,B Antennule and antenna. C Ventral view showing legs. a1: antennule, a2: antenna, ap: accessory processes

***Material examined***: ***Holotype***—1 ♀ (3.3 mm) (Reg. No. NSMT-Cr 31607), from *Chaunax abei* collected at a depth of 212 m off Suruga Bay, Japan (34°37′48.87″ N, 138°43′2.958″ E); coll. Y. Nishida and S. Ohtsuka on 20 October 2022.

***Description of holotype female***: Body, small, flattened, 2.8 times as long as wide (lateral processes not included), total body length 3.3 mm. Cephalothorax and first pedigerous somite fused forming cephalothorax. Cephalothorax, nearly as long as wide with anterior pair of large and posterior pair of small lobes, and two pairs of ventrolateral processes, posterior one located on either side of mouth appendages. Posteriormost part of the first pedigerous somite contributes to the neck. Third and fourth pedigerous somites fused forming cylindrical trunk, bearing two pairs of blunt, proximally fusiform processes with narrow stalk basally (one pair at proximo-lateral corner and other at disto-lateral corner, both directed postero-laterally). Genitoabdomen about twice wider than long. Caudal rami 1.2 times as long as wide, bearing one long spiniform and three short setiform elements. Egg sac multiseriate, elongate, not coiled (Fig. 1A); number of eggs in right and left egg sacs approximately 450 and 580, respectively.

Antennule (Figs. 1B, 3B, C, 5A) large, fleshy, swollen, with few small elements and small knob terminally. Antenna (Figs. 1C, 3D, 5A, B) uncinate, 3-segmented, with coxa and basis fused to form coxobasis and 2-segmented endopod, proximal endopodal segment typically produced into powerful, curved claw while distal endopodal segment represented by slender segment with five elements (Fig. 3E). Labrum protruding (Fig. 3A). Mandible (Fig. 3F) represented by falcate blade, bilaterally denticulated. Maxillule (Fig. 3G) vestigial, lobate, tipped with two apical spinules. Maxilla (Fig. 3H) 2-segmented; syncoxa unarmed; basis curved, slender, and attenuated process bearing row of teeth. Maxilliped (Fig. 3I) 3-segmented, armature of typical chondracanthid type; first segment robust, unarmed; second segment with a patch of minute spinules on inner edge; distal segment, small, ending in curved claw-like structure with small, subterminal hooklet on inner surface.

Two pairs of biramous legs (Fig. 5C); protopod unarmed; exopod and endopod 2-segmented, armed with setae. Other legs absent. Leg 1 (Fig. 4A), located near maxilliped; first exopodal segment with an outer seta; second segment with nine setae along free margin; first endopodal segment unarmed; second segment with seven setae along free margin. Leg 2 (Fig. 4B), located in neck region; first exopodal segment with an inner seta; second segment with four setae along distal margin; first endopodal segment unarmed; second segment with five setae along distal margin.

- ***Male***: Unknown.
- ***Color***: White (before fixation).
- ***Host***: Known only from *Chaunax abei* Le Danois, 1978 (Chaunacidae), the type host.
- ***Distribution***: Known only from the type locality.
- ***Etymology***: The specific name of the new species, ‘*nishidai*’, is dedicated to Mr. Yusuke Nishida (Hiroshima University) who found this enigmatic chondracanthid in the Suruga Bay, Japan. It is a noun in the genitive case.

### Genus *Kokeshioides* gen. nov.

*Type species*: *Kokeshioides surugaensis* **gen.** et **sp. nov.** by original designation.

***Diagnosis based on the adult female*** (**bold = key features**). Body small, flattened. Cephalothorax fused with first pedigerous somite forming cephalothorax. Cephalothorax much wider than long, with lobate anterior and posterior corners, folded ventrally. **Posteriormost part of the first pedigerous somite contributes to the neck**. Third and fourth pedigerous somites fused forming shield-like trunk, without lateral processes; trunk anteriorly narrower at transition to neck. Antennule fleshy, swollen, with many spinules. Antenna uncinate, without accessory process. Mandible elongate, falcate and bilaterally denticulate. Maxillule armed with one element. Maxilla 2-segmented; basis curved, slender, with attenuate process bearing row of teeth. Maxilliped 3-segmented, first segment robust, unarmed; second segment with a patch of minute spinules on inner edge; distal segment, small, ending in curved claw-like structure with small, subterminal hooklet on inner surface. **Legs 1 and 2, represented by pairs of acutely pointed processes**. **Other legs absent**. Genital double-somite, highly reduced with paired genital apertures on ventral surface. Caudal rami bearing 1 long and 3 short setae. Egg sac unknown.

***Etymology***: The generic name is derived from a Japanese traditional wooden toy called “Kokeshi” and the Latin suffix -*oides* meaning “like”. Gender masculine.

***Remarks***: The genus *Kokeshioides* **gen. nov.** is clearly distinguishable from all other known chondracanthid genera by the following combination of features: (1) body flattened, without any processess; (2) cephalothorax, much wider than long, with lobate corners, folded ventrally. (3) posteriormost part of the first pedigerous somite contributes to the neck; (4) mandible elongate; (5) legs 1 and 2 unique, represented by paired acutely pointed processes.

The presence of two pairs of highly modified spiniform legs makes *Kokeshioides* **gen. nov.** unique among all known chondracanthid genera. The genera *Humphreysia* and *Immanthe* show some similarity to *Kokeshioides* **gen. nov.**, especially in general body shape and the absence of trunk processes (see Table 1). In addition to the key diagnostic features of *Kokeshioides* **gen. nov.** (highly modified spiniform legs), *Humphreysia* can be differentiated from the latter by (1) antenna with vestigial tip (second endopodal segment) in *Humphreysia* (vs. without), (2) cephalic processes present in *Humphreysia* (vs. absent); (3) only one pair of biramous segmented legs with armature in *Humphreysia* (vs. two pairs of highly modified legs). *Immanthe* can be separated from the *Kokeshioides* by (1) presence of one pair of cephalic processes (vs. absent), and (2) rami of both legs, endopod smaller than exopod in *Immanthe* (vs. legs spiniform).

### ***Kokeshioides surugaensis*** gen. et sp. nov. (Figs. 6, 7, 8, 9, 10, 11)

**Fig. 6:**
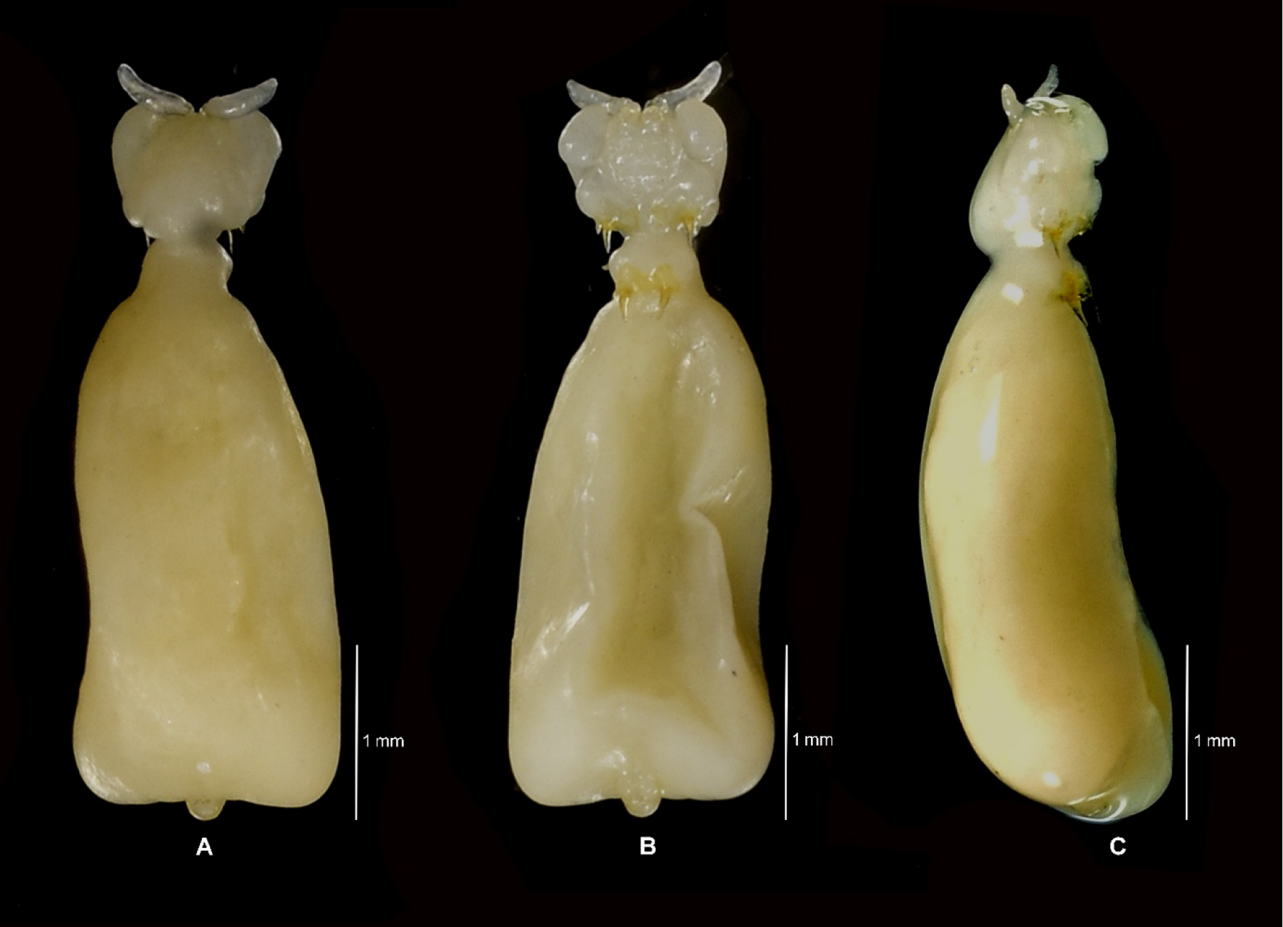
*Kokeshioides surugaensis* gen. et sp. nov., holotype female A Dorsal view. B Ventral view. C Lateral view

**Fig. 7:**
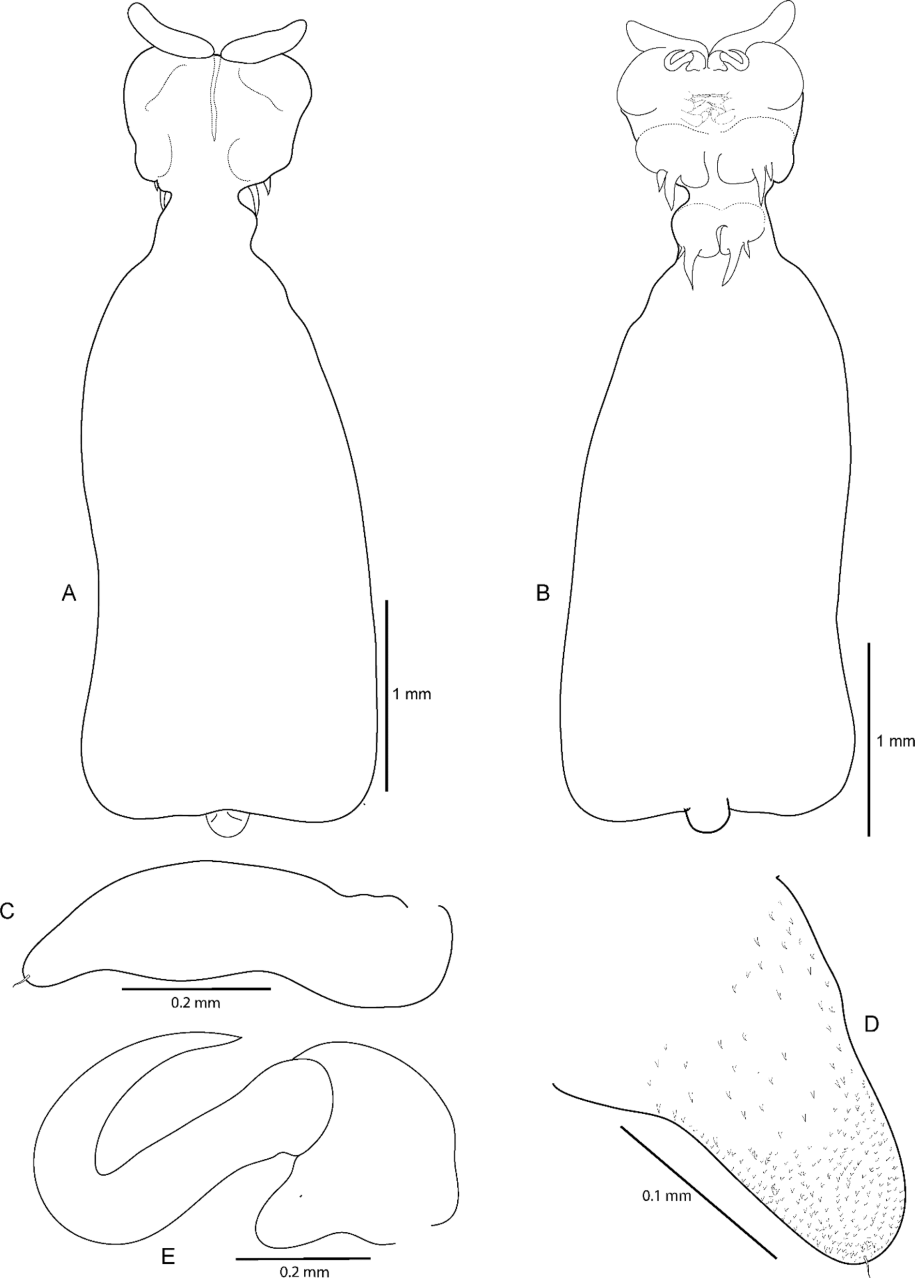
*Kokeshioides surugaensis* gen. et sp. nov. holotype female. A Dorsal view. B, ventral view. C–E Paratype female, C Antennule. D Antennule apex. E Antenna

**Fig. 8:**
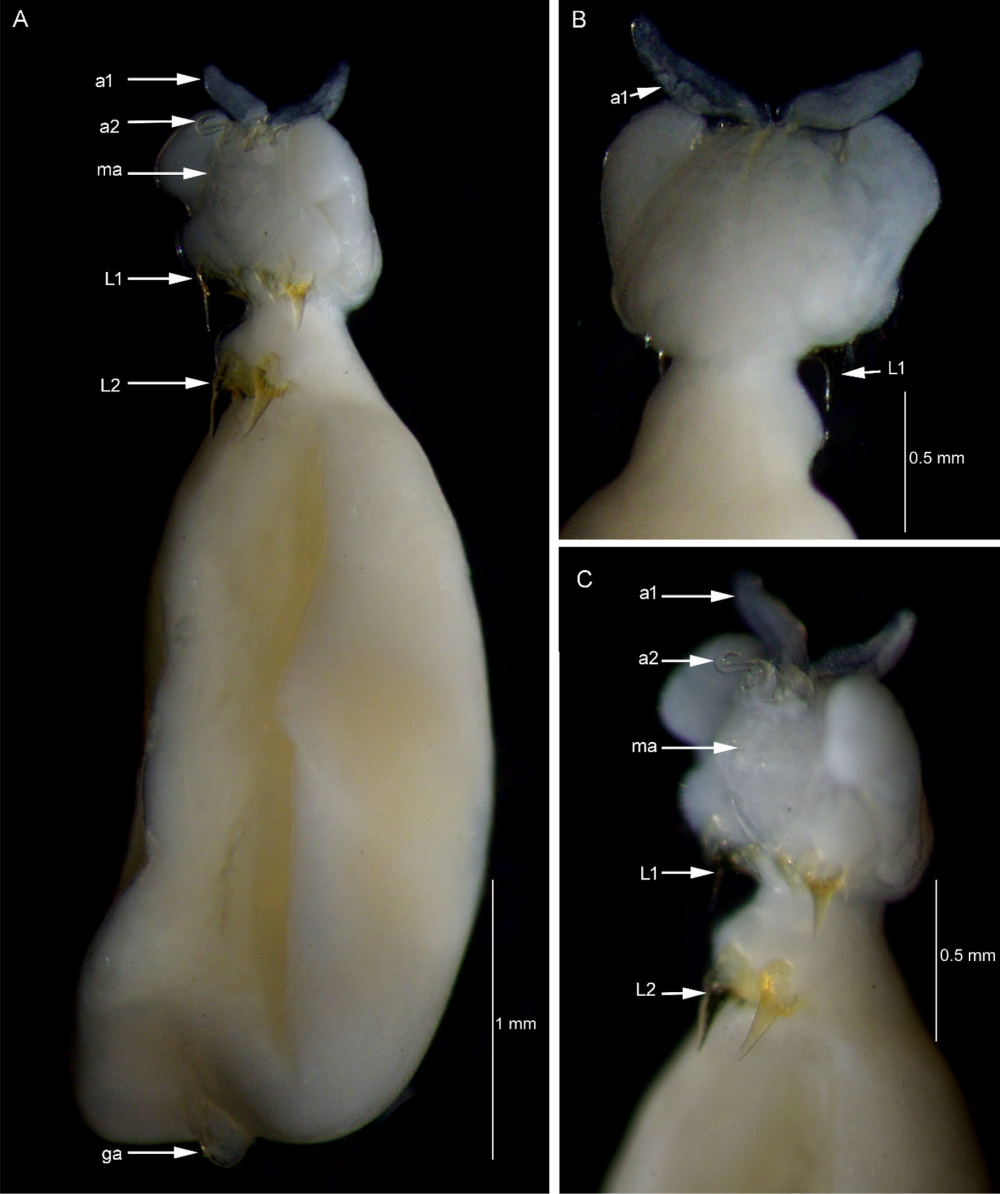
*Kokeshioides surugaensis* gen. et sp. nov., paratype female. A Habitus ventral view. B Cephalothorax, dorsal view. C Cephalothorax, ventral view. a1: antennule, a2: antenna, ma: mouth appendages, L1: leg 1, L2: leg 2. ga: genitoabdomen

**Fig. 9:**
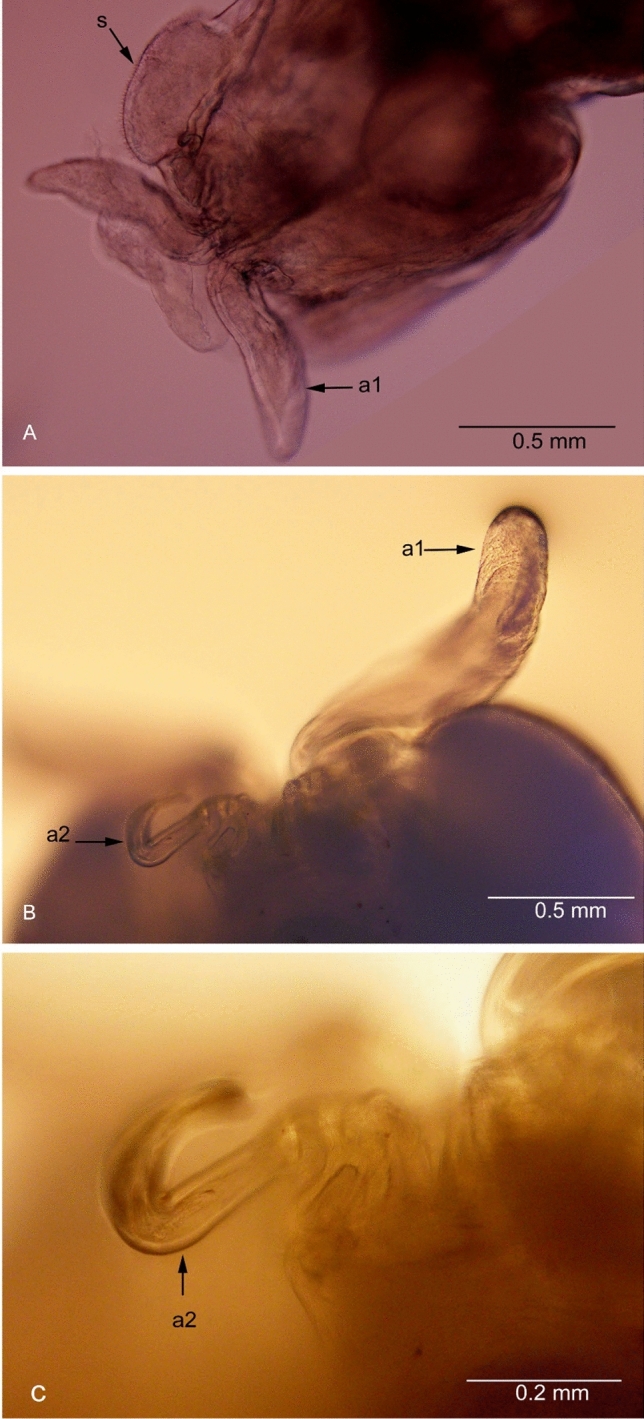
*Kokeshioides surugaensis* gen. et sp. nov., paratype female. A Cephalothorax, dorsal view. B Antennule and antenna. C Antenna. a1: antennule, a2: antenna, s: spinules

**Fig. 10:**
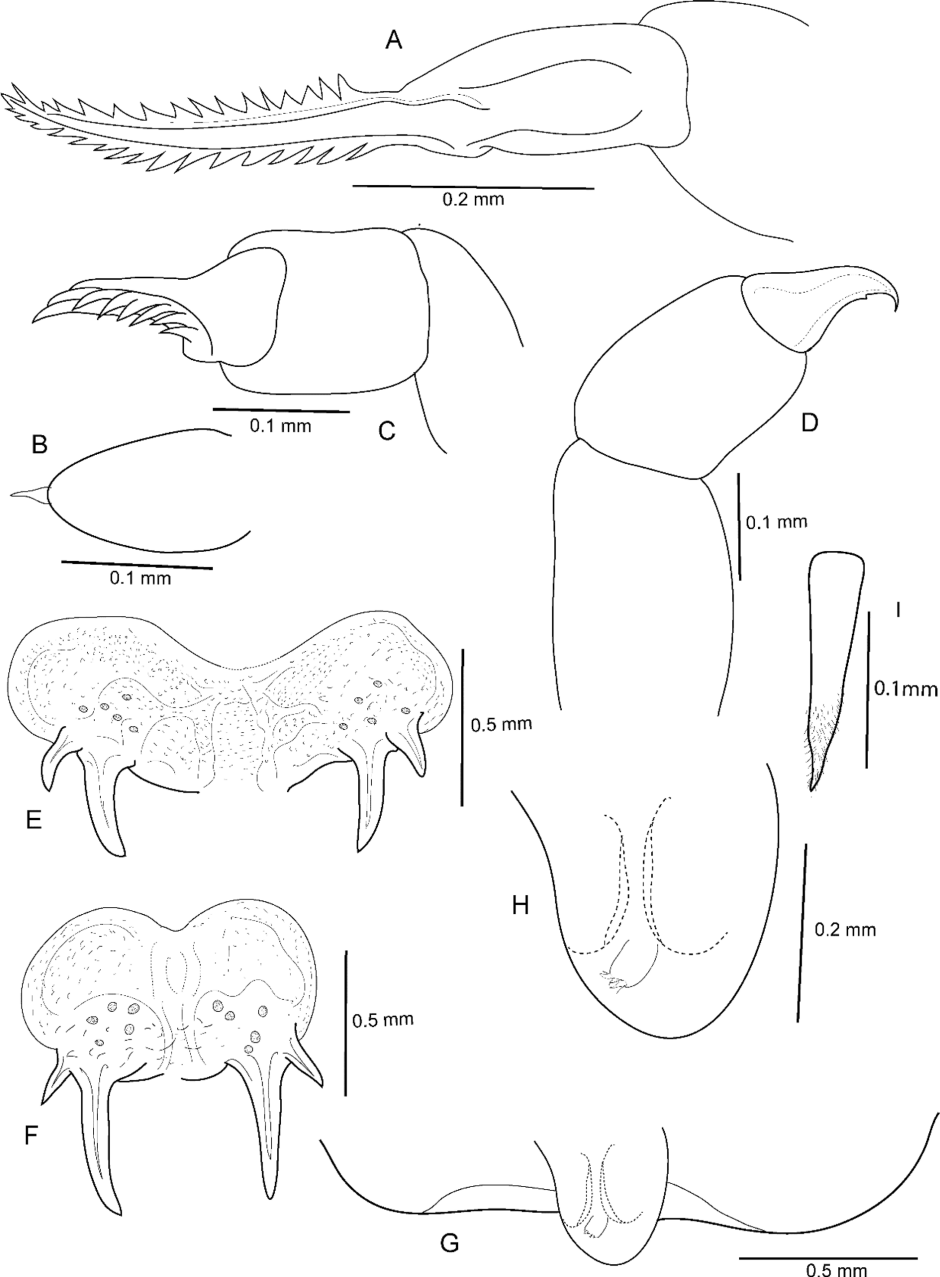
*Kokeshioides surugaensis* gen. et sp. nov. A Mandible. B Maxillule. C Maxilla. D Maxilliped. E Leg 1. F Leg 2. G,H Genitoabdomen. I Tip of caudal ramus showing caudal setae

**Fig. 11:**
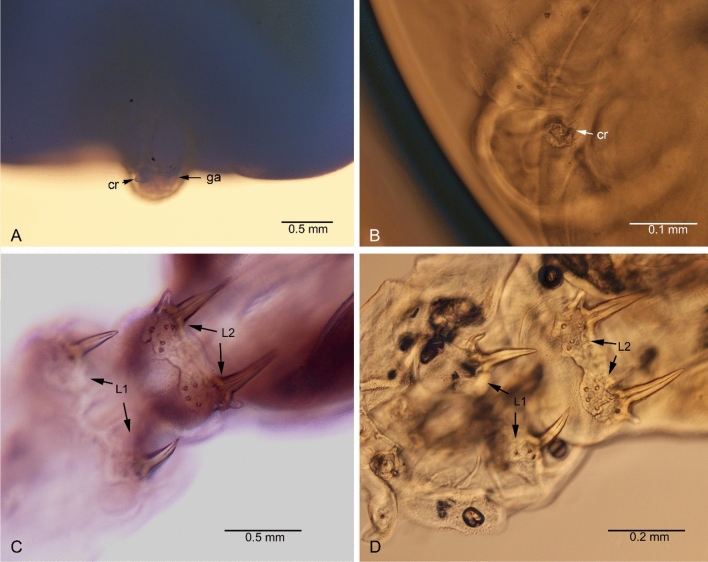
*Kokeshioides surugaensis* gen. et sp. nov. A,B Genitoabdomen. C,D Legs 1 and 2. cr: caudal rami, L1: leg 1, L2: leg 2, ga: genitoabdomen

***Material examined***: ***Holotype***—1 ♀ (4.1 mm) (Reg. No. NSMT-Cr 31608), from *Setarches longimanus* (Alcock, 1894) (Setarchidae), collected at a depth of 212 m off Suruga Bay, Japan (34°37′48.87″ N, 138°43′2.958″ E); coll. S. Ohtsuka on 20 October 2022. ***Paratype***—1 ♀ (4.0 mm) (Reg. No.NSMT-Cr 31609); same data as holotype.

***Description of holotype female***. Body (Figs. 6, 7, 8), small, flattened, 2.7 times as long as wide, widest in posterior part, total body length, 4.0–4.1 mm. Cephalothorax consisting of cephalothorax and first pedigerous somite. Cephalothorax, 1.4 times as wide as long, with lobate extensions at anterior and posterior corners, folded ventrally; lateral surface of dorsal shield armed with many spinules. Posteriormost part of the first pedigerous somite contributes to the neck. Third and fourth pedigerous somites fused, forming trunk, without lateral processes as expressed in *Avatar nishidai* **gen. et sp. nov.**; trunk tapering anteriorly towards transition with neck (Fig. 8). Genitoabdomen (Figs. 8A, 10G–I, 11A, B) highly reduced with paired genital apertures on ventral surface, carrying pair of ventrally folded caudal rami, bearing 1 long and 3 short setae. Egg sacs not observed.

Antennule (Figs. 7C, D, 8, 9A, B, D) fleshy, with large, swollen basal portion and small setose terminal part. Antenna (Figs. 7E, 8A, C, 9B) uncinate, 2-segmented, basis without any process; endopod modified into medially-curved hook. Mandible (Fig. 10A) elongate, falcate blade, bilaterally denticulate. Maxillule (Fig. 10B) vestigial, lobate, armed with one minute element. Maxilla (Fig. 10C) 2-segmented, terminal segment represented by curved, slender, attenuated process bearing row of teeth. Maxilliped (Fig. 10D) 3-segmented, first segment robust, unarmed; second segment with a patch of minute spinules on inner edge; distal segment, small, ending in curved claw-like structure with small, subterminal hooklet on inner surface.

Two pairs of biramous, highly modified legs (Figs. 6, 7B, 8, 10E, F, 11C, D; basis broad, plate-like, surface slightly rough, with patches of minute pustules. Rami modified into acutely pointed processes, movable at base (arrowed in Fig. 11C, D). Other legs absent. Leg 1 (Figs. 10E, 11C, D) located posterior to mouth appendages, basis unarmed; exopod, shorter than endopod (0.6 times as long as endopod). Leg 2 (Figs. 10F, 11C, D) located in neck region, basis slightly narrower than that of leg 1; exopod shorter than endopod (0.4 times as long as endopod).

- ***Male***: Unknown.
- ***Color***: White (before fixation).
- ***Host***: Known only from *Setarches longimanus* (Alcock, 1894) (Setarchidae).
- ***Distribution***: Known only from the type locality.
- ***Etymology***: The specific name is derived from the type locality, Suruga Bay, Japan. It is in the nominative singular, gender masculine.

## Discussion

The family Chondracanthidae was thoroughly revised by Ho [25], who excluded 12 old genera and provided a key to the 30 valid genera recognized at that time. Twelve genera were added subsequently by various authors which Ho [26] included in his revised key. Three genera were subsequently synonymised [27]. After the revision of Ho [26] eight new genera have been established, the latest addition of which being the discovery of *Ttetaloia* Uyeno and Nagasawa, 2012 from Japan and the three previously excluded genera (viz. *Immanthe* Leigh-Sharpe, 1934, *Lernaeosolea* Wilson, 1944, and *Pharodes* Wilson, 1935) were restored, based on unique combinations of generic characters [2, 6, 26, 27]. With the addition of the two new genera in the presently reported study, *Avatar* **gen. nov.** and *Kokeshioides* **gen. nov.**, the family Chondracanthidae currently includes 52 valid genera.

Many chondracanthid genera possess usually paired ventrolateral processes on the 3 or 4 pedigerous somites; for instance, members of the genus *Chondracanthus* have well-developed trunk processes, and their number may vary in different species. Our observation of *Chondracanthus kabatai* Aneesh, Helna, and Kumar, 2020 reveals that these ventrolateral processes contain oviducts, providing space for the maturing oocytes, and thereby enhancing the fecundity. The majority of chondracanthid genera possess an uncinate, strongly prehensile antenna; few others have a bifurcate, trifurcate or clavate (non-uncinate type) antenna and some relatively primitive chondracanthid genera such as *Avatar* **gen. nov.**, *Humphreysia*, *Immanthe*, *Juanettia* etc., possess the distal endopodal segment, represented by the atrophied tip of the antenna as a slender segment with 2–6 apical elements [28, 29]. The structure seems to have been lost during evolution. For instance, it is totally absent in highly advanced genera such as *Acanthochondria*, *Chondracanthus*, *Kokeshioides* **gen. nov.** etc. In contrast, the morphology of the mouth appendages seems to be relatively constant within the family, suggesting that most species share a similar feeding mode [21, 25, 26].

Various evolutionary trends can be observed in the development of legs, especially legs 1 and 2 in the family. Legs are highly modified or rudimentary in females chondracanthids and vary in different genera: only one biramous 2-segmented leg is present in primitive chondracanthids such as *Juanettia*, *Auchenochondria* Dojiri and Perkins, 1979 and *Humphreysia*; two pairs of unmodified, biramous legs are present in *Avatar* **gen. nov.**; uniramous or unilobate or bilobate legs are present in remaining chondracanthid genera; and legs are totally absent in *Apodochondria* Ho and Dojiri, 1988 (see Ho [25, 26]). The highly modified, spiniform legs in *Kokeshioides* **gen. nov.** can be considered the most advanced state in the Chondracanthidae. The segmentation of the legs is clear in the relatively primitive genera such as *Cryptochondria* Izawa, 1971, *Juanettia* and *Rhynchochondria* Ho, 1967. Between these most advanced and primitive conditions, a wide variety of intermediate conditions can be seen: the segmentation of the rami of the legs is lost in some species of *Protochondria* Ho, 1970, reduction in size of the rami of legs in *Brachiochondrites* Markevich, 1940, *Blias*, *Chondracanthodes* Wilson, 1932 or just in the size of the endopod in *Humphreysia* (see Kabata [30]). Along with the size reduction, the number and size of setae are also reduced in *Pseudacanthocanthopsis* Yamaguti and Yamasu, 1959 and some species of *Protochondracanthus* Kirtisinghe, 1950, and seta are totally absent in many advanced genera in *Blias*, *Chondracanthus*, *Chondracanthodes*, *Pseudacanthocanthopsis*, *Pseudoblias* Heegaard, 1962, *Acanthochondria*, and *Acanthochondrites* Oakley, 1930. The legs will be either totally absent or highly modified, represented by setae or spiniform processes during the course of evolution (in *Kokeshioides* **gen. nov.**) (see Kabata [30]). Throughout the evolution of the family, legs might have been reduced into functionless elements or modified new functional grasping organs.

## Conclusions

New material collected from two different species of deep-sea fishes of Suruga Bay, Japan were found to be different from all other known chondracanthid genera, and based on the clear morphological features we described two new monotypic genera. Accordingly, we described *Avatar nishidai* **gen.** et **sp. nov.** from *Chaunax abei* Le Danois, 1978 (Chaunacidae) and *Kokeshioides surugaensis* **gen.** et **sp. nov.** from *Setarches longimanus* (Alcock, 1894) (Setarchidae). By the description of two new genera in the presently reported study, the family Chondracanthidae currently includes 52 valid genera. Among the described genera *Avatar* **gen. nov.** seems to be very primitive, while *Kokeshioides* **gen. nov.** is highly advanced. The deduced evolutionary history of chondracanthid genera is also discussed in the present paper.

## Data Availability

The type materials are deposited in the National Museum of Nature and Science, Tsukuba, Japan.
